# A Comparative Study of Assay Performance of Commercial Hepatitis E Virus Enzyme-Linked Immunosorbent Assay Kits in Australian Blood Donor Samples

**DOI:** 10.1155/2016/9647675

**Published:** 2016-11-07

**Authors:** Ashish C. Shrestha, Robert L. P. Flower, Clive R. Seed, Susan L. Stramer, Helen M. Faddy

**Affiliations:** ^1^Research and Development, Australian Red Cross Blood Service, Brisbane, QLD, Australia; ^2^School of Medicine, The University of Queensland, Brisbane, QLD, Australia; ^3^Medical Services, Australian Red Cross Blood Service, Perth, WA, Australia; ^4^American Red Cross, Gaithersburg, MD, USA

## Abstract

Hepatitis E virus (HEV) is transfusion-transmissible and therefore poses a risk to blood transfusion safety. Seroprevalence studies are useful for estimating disease burden and determining risk factors. Considerable variability in the sensitivity of HEV antibody detection assays exists. This study aimed to compare the performances of commercially available HEV enzyme-linked immunosorbent assays (ELISA) in Australian blood donor samples. Plasma samples that tested positive (*n* = 194) or negative (*n* = 200) for HEV IgG (Wantai HEV IgG ELISA) were selected. Of the 194 HEV IgG positive samples, 4 were positive for HEV IgM (Wantai HEV IgM ELISA). All samples were tested with the MP Diagnostics: HEV IgG ELISA, total (IgG, IgM, and IgA) HEV antibody ELISA, and HEV IgM ELISA. Of the 194 Wantai HEV IgG positive samples, 92 (47%) tested positive with the MP Diagnostics HEV IgG ELISA (*κ* = 0.47) and 126 (65%) with MP Diagnostics total HEV antibody assay (*κ* = 0.65). There was poor agreement between Wantai and MP Diagnostics HEV IgM assays. This study demonstrated poor agreement between the assays tested. These observations are consistent with previous reports demonstrating significant variability between HEV ELISAs, highlighting that results of HEV serology should be interpreted with caution.

## 1. Introduction

Hepatitis E virus (HEV) is a nonenveloped, RNA virus, classified in the genus* Hepevirus* of the Hepeviridae family [1]. There are 4 genotypes of HEV [1–4], representing a single serotype, which infect humans [2]. This classification into genotypes is based on variation in the nucleotides within open reading frame-2 (ORF-2) [3, 4]. HEV was first observed under immune electron microscopy in stool samples from a volunteer experimentally infected with non-A, non-B hepatitis [5]. Isolation of cDNA identified this virus as being different from hepatitis A [6] and facilitated the development of serological assays for HEV.

HEV causes self-limited acute phase disease with known cases of chronic hepatitis [7]. The incubation period on average is 40 days [8]. Clinical features include anorexia, nausea, vomiting, diarrhoea, epigastric pain, fever, jaundice, elevation of serum transaminase, and hepatomegaly [5, 7, 9–11]. Chronic HEV infections have been reported in solid-organ transplant recipients [12] and in immune suppressive conditions [13, 14]. A case fatality rate of 0.5–4% has been reported in developing countries [7], which is as high as 10–25% in pregnant women during the third trimester [2, 15, 16].

HEV is transfusion-transmissible and causes chronic infections in immunocompromised individuals [17]. The risk of transfusion-transmission from a donor with asymptomatic viraemia can be identified through the detection of HEV RNA. However, the detection of HEV antibodies provides useful information on the immune status or stages of HEV infection in blood donors and may assist with the identification of risk factors for exposure. Seroprevalence is also important for assessing the overall disease burden in a population, and studies have shown that HEV exposure in blood donors varies widely between geographical regions [18, 19]. For example, 6% of Australian blood donors have been shown to be HEV IgG positive, while 52% of donors in southwestern France were HEV IgG positive with the same assay [20, 21].

Serology-based HEV tests for the detection of viral-specific antibodies include the detection of HEV IgG, HEV IgM, and HEV IgA in serum or plasma. Antibody testing assays are generally based on the detection of antibodies against epitopes of the gene products from ORF2 and ORF3 [22]. Many enzyme immunoassays with antigens derived from one HEV genotype are able to detect antibodies against a different genotype [23]. Detection of HEV IgG in an individual indicates a previous HEV infection. This antibody may persist in an infected individual for more than 12 years [24]. The acute phase of HEV infection can be detected by the detection of HEV IgM. This class of antibody is detectable after the onset of acute hepatitis and can last for up to 6 months following infection [25].

Studies with different commercial HEV IgG enzyme immunoassays have shown variability in sensitivity [26–28]. A study using anti-HEV reference serum (from the World Health Organisation) and including known HEV cases has shown 98% seropositivity with the Wantai IgG assay compared to 56% with the Genelabs IgG assay [27]. In a Korean study, HEV IgG seroprevalence was measured to be 23.1% with the Wantai assay, compared to 14.3% with the Genelabs assay [29]. Moreover, a study in HEV infected individuals has shown positivity of 83.3%, 100%, and 96.7% with the MP Diagnostics assay, Axiom Diagnostics assay (developed by Wantai), and Mikrogen assay, respectively [30]. Seroprevalence determined with different assays therefore needs to be interpreted with caution. Evaluation of HEV IgM commercial assays has also shown variability in sensitivity and specificity [31]. Given the importance of reliable seroprevalence estimates, this study aimed to compare the performances of commercially available HEV antibody detection assays (IgG and/or IgM) using a panel of Australian blood donor samples, made up of preselected positive and negative samples by one widely used assay.

## 2. Materials and Methods

### 2.1. Samples

Plasma samples from individual donors (*n* = 394) selected from a previous HEV seroprevalence study [20] were included in this study. These included samples (*n* = 194) that tested positive for HEV IgG with the Wantai HEV IgG ELISA (Beijing Wantai Biological Pharmacy, Beijing, China). These positive samples were all of the HEV IgG positive samples obtained from the previous seroprevalence study, which included 3,237 donors randomly selected for sex and age group [20]. Of the HEV IgG positive samples, 4 were also positive for HEV IgM with Wantai HEV IgM ELISA. In addition, age-matched negative samples (*n* = 200) were also sourced from the same seroprevalence study. Blood samples were collected in EDTA tubes (BD Vacutainer® Whole Blood Collection tube with spray-coated K2EDTA 6 mL, Becton Dickinson, Plymouth, UK), centrifuged at 1,258 g for 5 minutes and stored at −20°C until testing. Convenience samples no longer required after routine viral screening were utilised for this study and all samples were collected between August and September, 2013. The age of the donor was obtained from Blood Service records. This study was approved by Blood Service Human Research Ethics Committee.

### 2.2. Sample Testing: Wantai HEV ELISAs

The above-selected samples were tested for HEV IgG with the Wantai HEV IgG ELISA (Beijing Wantai Biological Pharmacy Enterprise Co., Ltd.). Samples reactive for HEV IgG were tested for HEV IgM with the Wantai HEV IgM ELISA (Beijing Wantai Biological Pharmacy Enterprise Co., Ltd.). Samples were tested as per the manufacturer's instructions and absorbance was measured using a Hybrid Multimode Microplate Reader (BioTek Instruments, Inc., Winooski, USA) at 450 nm. Samples initially reactive for HEV IgG or HEV IgM were retested in duplicate with the respective assay and considered positive if reactive at least twice. After testing, samples were aliquoted into microtubes (Axygen Inc., USA) and stored at −20°C prior to testing with secondary commercial assays.

The Wantai HEV IgG assay is based on a recombinant HEV PE2 protein containing 211 amino acids of ORF2 derived from HEV genotype 1 [26, 27]. Sensitivity and specificity of the HEV IgG assay have been shown to be 97.96% and 99.6%, respectively [32, 33]. The Wantai HEV IgM assay is also based on a recombinant protein derived from HEV ORF2 [34]. Sensitivity of HEV IgM assay has been shown to be 97.10% [34]. Both the assays required 10 *μ*L of sample, which was diluted with diluent (1 : 11) [32, 34].

### 2.3. Sample Testing: MP Diagnostics ELISAs

The above-selected samples were tested in singlet for HEV IgG with the MP Diagnostics HEV ELISA (MP Biomedicals Asia Pacific, Singapore), total (IgG, IgM, and IgA) HEV antibody with the MP Diagnostics HEV ELISA 4.0 (MP Biomedicals); and HEV IgM with the MP Diagnostics HEV IgM ELISA 3.0 (MP Biomedicals). Samples were tested as per the manufacturer's instructions and absorbance was measured using a Hybrid Multimode Microplate Reader (BioTek Instruments, Inc.) at 450 nm. Samples initially reactive with each assay were retested in duplicate with the same assay and considered positive if reactive at least two out of three times.

The MP Diagnostics HEV IgG assay uses three recombinant proteins, consisting of 42-amino acid sequence derived from ORF2 of genotype 2, 33-amino acid sequence from ORF3 of genotype 3, and ORF3 sequence from genotype 1 [26]. The assay has a reported sensitivity of 98% and specificity of 97% [35]. The assay required 10 *μ*L of sample and was diluted with diluent (1 : 21).

MP Diagnostic HEV ELISA 4.0 detects IgG, IgM, and IgA antibodies. The assay uses highly conserved HEV ORF2.1 antigen, which is able to detect all antibody isotypes [36]. The test required 20 *μ*L of sample and was diluted with diluent (1 : 50). The assay has a reported sensitivity of 99.2% and specificity of 99.2% [35].

MP Diagnostic HEV IgM ELISA is based on genotype 1 and 2 antigens derived from ORF2 and ORF3 [37]. The assay has a reported sensitivity of 98% and specificity of 96.7% [35]. The assay used 10 *μ*L of sample and was diluted with diluent (1 : 21).

### 2.4. Data Analysis

Sample to cut-off ratio was calculated, and results were interpreted based on criteria from the manufacturers' instructions. Concordance between assays was determined by calculating Kappa (*κ*) correlation, which measures the agreement between two assays, using IBM SPSS Statistics 23 (IBM Centre, NSW, Australia).

## 3. Results

**Figure 1:**
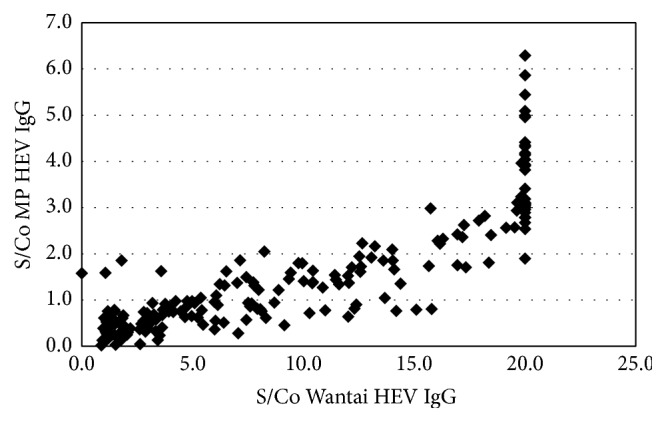
S/Co Wantai HEV IgG versus MP Diagnostics HEV IgG.

**Figure 2:**
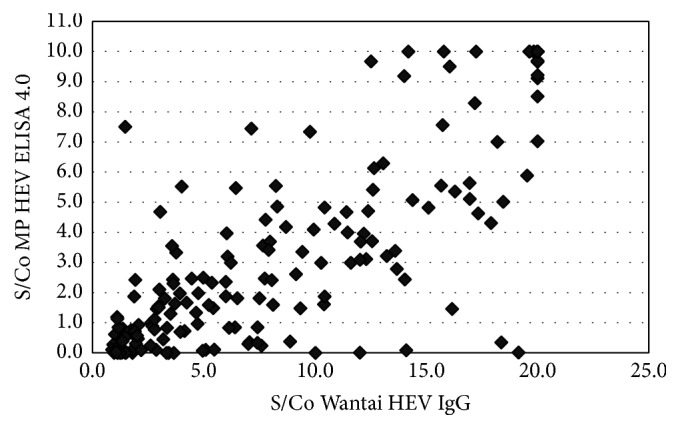
S/Co Wantai HEV IgG versus MP Diagnostics HEV ELISA 4.0 (IgG, IgM, and IgA).

Of the 194 Wantai HEV IgG reactive samples, 92 were reactive with the MP Diagnostics HEV IgG ELISA. One of the 200 negative samples with the Wantai HEV IgG assay tested positive with MP Diagnostics HEV IgG ELISA. There was a poor agreement between these assays (*κ* = 0.47) (Table 1, Figure 1). However, the agreement between MP Diagnostics total HEV antibody assay and Wantai HEV IgG was higher (*κ* = 0.65) with 126/194 testing positive (Table 2, Figure 2). All the Wantai HEV IgG negative samples were also negative with MP Diagnostics total HEV antibody assay. Of the 4 Wantai HEV IgM positive samples, none tested positive for HEV IgM on the MP Diagnostics HEV IgM ELISA (Table 3). All Wantai HEV IgM positive samples were positive with MP Diagnostics total HEV antibody assay.

**Figure 3:**
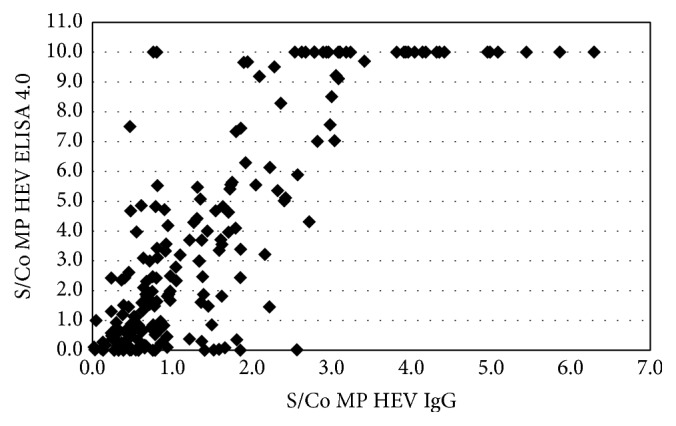
S/Co MP Diagnostics HEV IgG versus MP Diagnostics HEV ELISA 4.0 (IgG, IgM, and IgA).

Comparing the test results between the MP Diagnostics total HEV antibody ELISA and MP Diagnostics HEV IgG ELISA, 82 of 126 (88.17%) tested positive with the latter (*κ* = 0.65). However, 11 of the samples that tested negative with MP Diagnostics total HEV antibody ELISA were positive with MP Diagnostics HEV IgG ELISA (Table 4, Figure 3). Of these, 10 samples were positive with Wantai HEV IgG ELISA.

## 4. Discussion

HEV is a causative agent of acute hepatitis. The majority of HEV cases in developed countries are in travellers returning from developing countries endemic for HEV [25]; however, autochthonous HEV related to zoonotic transmission [2] and transfusion-transmission [17] have also been reported. HEV serological assays have allowed seroprevalence studies, which provide useful surveillance data on the distribution of this virus, and have also assisted with identifying risk factors for exposure to HEV. However, studies have shown variability in estimates with different commercial assays [21, 28], and the results presented herein are consistent with such findings.

In this study, a poor concordance of test results between the two tested commercial HEV IgG ELISAs was observed. Only 47% (92/194) of Wantai HEV IgG positive samples were positive with the MP Diagnostics HEV IgG (*κ* = 0.47). This observation is similar to a Korean study, which also compared Wantai and Genelabs (now MP Diagnostics) HEV IgG assays (*κ* = 0.31) [29]. One of the samples negative with Wantai HEV IgG assay was positive with the MP Diagnostic HEV IgG assay (0.50%), similar to an observation in a French study (0.69%) using a Fortress Diagnostics assay that uses Wantai recombinant proteins [26]. Previous studies have shown that the Wantai HEV IgG ELISA is one of the most sensitive commercial assays available for the detection of HEV IgG [27, 29, 30].

Our study also showed a higher agreement between the Wantai HEV IgG and MP Diagnostic HEV total antibody assay (*κ* = 0.65). The total antibody ELISA is more recently developed (compared to the MP Diagnostics HEV IgG assay) incorporating an improved antigen with the ability to detect total antibodies (IgG, IgM, and IgA) against all HEV genotypes [36]. It is possible that the Wantai HEV IgG could have given nonspecific results, but the majority of Wantai HEV IgG negative samples still tested negative with the MP Diagnostic HEV IgG assay (199 of 200). In addition, there was also nonconcordance between MP Diagnostic total and IgG assays. The proportion of samples positive with the MP Diagnostics IgG assay compared to the proportion positive with the MP Diagnostics total assay was unexpected (88%) and therefore questions the performance of the IgG assay assuming that the samples represented true positives.

Comparison of the Wantai HEV IgM and MP Diagnostics HEV IgM assays also showed poor agreement between these assays. A prior study has shown good specificity of the MP Diagnostic HEV IgM assay (99.5%) [37]. However, in our study, one HEV IgM positive sample with the MP Diagnostics HEV IgM assay was negative with MP Diagnostic total antibody assay. All four samples positive with Wantai HEV IgM assay were also positive with MP Diagnostic total antibody assay, demonstrating agreement between these assays.

The observed variability in assay performance could be explained by differences in recombinant proteins, assay formats, or other components (e.g., diluents) used in each assay, as well as sample selection given they were primarily preselected Wantai IgG-positive. Additional studies are required to elucidate the exact mechanism; however, it is clear that a “gold standard” for HEV antibody detection is desperately needed. The validity of serological assays for use in a particular study should be assessed prior to their use, and control samples from individuals diagnosed with HEV should be included wherever possible. Given that neither the infection history nor the exact serostatus (positive or negative based on confirmatory assays) of the samples was known, sensitivity and specificity of these assays could not be assessed in the present study. Thus, the findings of this study should be interpreted considering this limitation. Further studies including pedigreed seropositive/negative samples or those from individuals with a known history of HEV infection are clearly required.

## 5. Conclusion

In this study, a poor concordance of test results between the Wantai and MP Diagnostics HEV ELISAs was observed. Variability in results was likely due to differences in antigens, assay format, or other components used in each assay, as well as the fact that assumed seropositive samples were primarily preselected Wantai IgG-positive samples. These observations are consistent with previous reports demonstrating significant variability between HEV ELISAs, highlighting that due caution is required when interpreting the results of HEV serology. There is still a need for the development of sensitive, specific, and cost-effective HEV antibody assays, including confirmatory tests, to aid in estimating disease burden and determining risk factors for HEV exposure.

## Figures and Tables

**Table 1:** Comparison of test results between the Wantai HEV IgG ELISA and MP Diagnostics HEV ELISA (IgG).

Wantai (HEV IgG)	MP Diagnostics (HEV IgG)		Total
	Positive	Negative	
Positive	92 (47.4%)	102	194
Negative	1	199 (99.5%)	200
Total	93	301	394

**Table 2:** Comparison of test results between the Wantai HEV IgG ELISA and MP Diagnostics HEV ELISA 4.0 (IgG, IgM, and IgA).

Wantai (HEV IgG)	MP Diagnostics (HEV IgG, IgM, and IgA)		Total
	Positive	Negative	
Positive	126 (64.94%)	68	194
Negative	0	200 (100%)	200
Total	126	268	394

**Table 3:** Comparison of test results between the Wantai HEV IgM ELISA and MP Diagnostics HEV IgM ELISA 3.0.

Wantai (HEV IgM)	MP Diagnostics (HEV IgM)		Total
	Positive	Negative	
Positive	0	4	4
Negative	5	385 (98.7%)	390
Total	5	389	394

**Table 4:** Comparison of test results between the MP Diagnostics HEV ELISA (IgG) and MP Diagnostics HEV ELISA 4.0 (IgG, IgM, and IgA).

MP Diagnostics (HEV IgG)	MP Diagnostics (HEV IgG, IgM, and IgA)		Total
	Positive	Negative	
Positive	82 (88.17%)	11	93
Negative	44	257 (85.38%)	301
Total	126	268	394
